# Bimodal DNA self-origami material with nucleic acid function enhancement

**DOI:** 10.1186/s12951-024-02296-9

**Published:** 2024-01-26

**Authors:** Songlin He, Haotian Deng, Peiqi Li, Qinyu Tian, Yongkang Yang, Jingjing Hu, Hao Li, Tianyuan Zhao, Hongkun Ling, Yin Liu, Shuyun Liu, Quanyi Guo

**Affiliations:** 1 Institute of Orthopedics, First Medical Center, Chinese PLA General Hospital; Beijing Key Laboratory of Regenerative Medicine in Orthopedics; Key Laboratory of Musculoskeletal Trauma and War Injuries PLA, 28 Fuxing Road, Haidian District, Beijing, 100853 China; 2 School of Medicine, Nankai University, Tianjin, 300071 China; 3 Department of Gastroenterology, the Second Medical Center and National Clinical Research Center of Geriatric Diseases, 28 Fuxing Road, Haidian District, Beijing, 100853 China; 4 Nankai University Eye Institute, Nankai University, Tianjin, 300071 China

**Keywords:** DNA nanostructure, DNA nanoflower, DNA nanonet, Cell recruitment, Nucleic acid delivery

## Abstract

**Background:**

The design of DNA materials with specific nanostructures for biomedical tissue engineering applications remains a challenge. High-dimensional DNA nanomaterials are difficult to prepare and are unstable; moreover, their synthesis relies on heavy metal ions. Herein, we developed a bimodal DNA self-origami material with good biocompatibility and differing functions using a simple synthesis method. We simulated and characterized this material using a combination of oxDNA, freeze–fracture electron microscopy, and atomic force microscopy. Subsequently, we optimized the synthesis procedure to fix the morphology of this material.

**Results:**

Using molecular dynamics simulation, we found that the bimodal DNA self-origami material exhibited properties of spontaneous stretching and curling and could be fixed in a single morphology via synthesis control. The application of different functional nucleic acids enabled the achievement of various biological functions, and the performance of functional nucleic acids was significantly enhanced in the material. Consequently, leveraging the various functional nucleic acids enhanced by this material will facilitate the attainment of diverse biological functions.

**Conclusion:**

The developed design can comprehensively reveal the morphology and dynamics of DNA materials. We thus report a novel strategy for the construction of high-dimensional DNA materials and the application of functional nucleic acid–enhancing materials.

**Graphical Abstract:**

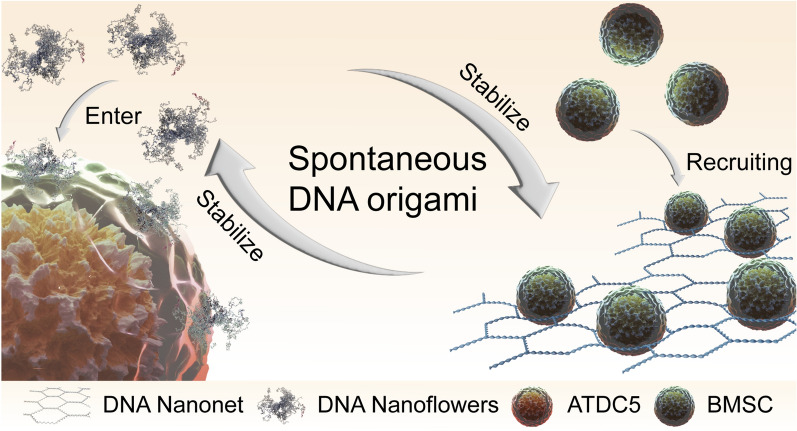

**Supplementary Information:**

The online version contains supplementary material available at 10.1186/s12951-024-02296-9.

## Background

DNA nanomaterials are prepared based on the Watson–Crick base pairing principle and exhibit high biocompatibility and programmability [1]. Over the past few decades, DNA nanomaterials have found widespread applications in various biological and medical fields, including cell recruitment, organoid construction, protein capture, and signal detection [2–15]. By manipulating DNA molecules, we can construct DNA nanomaterials with diverse nanostructures to achieve distinct functions [16–18]. DNA nanomaterials can be categorized into two-dimensional (2D) and three-dimensional (3D) nanostructures. 2D DNA nanomaterials encompass DNA aptamers [19] and DNA nanonets (DNNs) [20], whereas 3D DNA nanomaterials consist of 3D DNA cross-linking networks [7] and tetrahedral nucleic acids [21]. High-dimensional DNA materials can perform various complex biological functions, such as cell capture [7, 8], gel construction [9], signal sensing [10], cell protection [11], targeted drug delivery [12–14], and molecular power motors [15]. These functions are highly significant in the field of biomedical tissue engineering.

Currently, DNA origami stands as a crucial method for constructing DNA materials [23–25]. This innovative technique facilitates the folding of long DNA chains into 3D nanomaterials with diverse shapes, including DNA nanoflowers (DNFs). DNFs have proven effective in imaging and the targeted delivery of drugs to cells [26–29]. However, the construction of intricate DNA materials often relies on the use of heavy metal ions, such as Co and Mn [30–32]. This approach is complex, unstable, and challenging to control. Furthermore, heavy metal ions may induce cytotoxicity, which limits the medical application of the DNA material. Consequently, there is a need for a simple DNA construction method capable of overcoming these drawbacks.

**Scheme 1:**
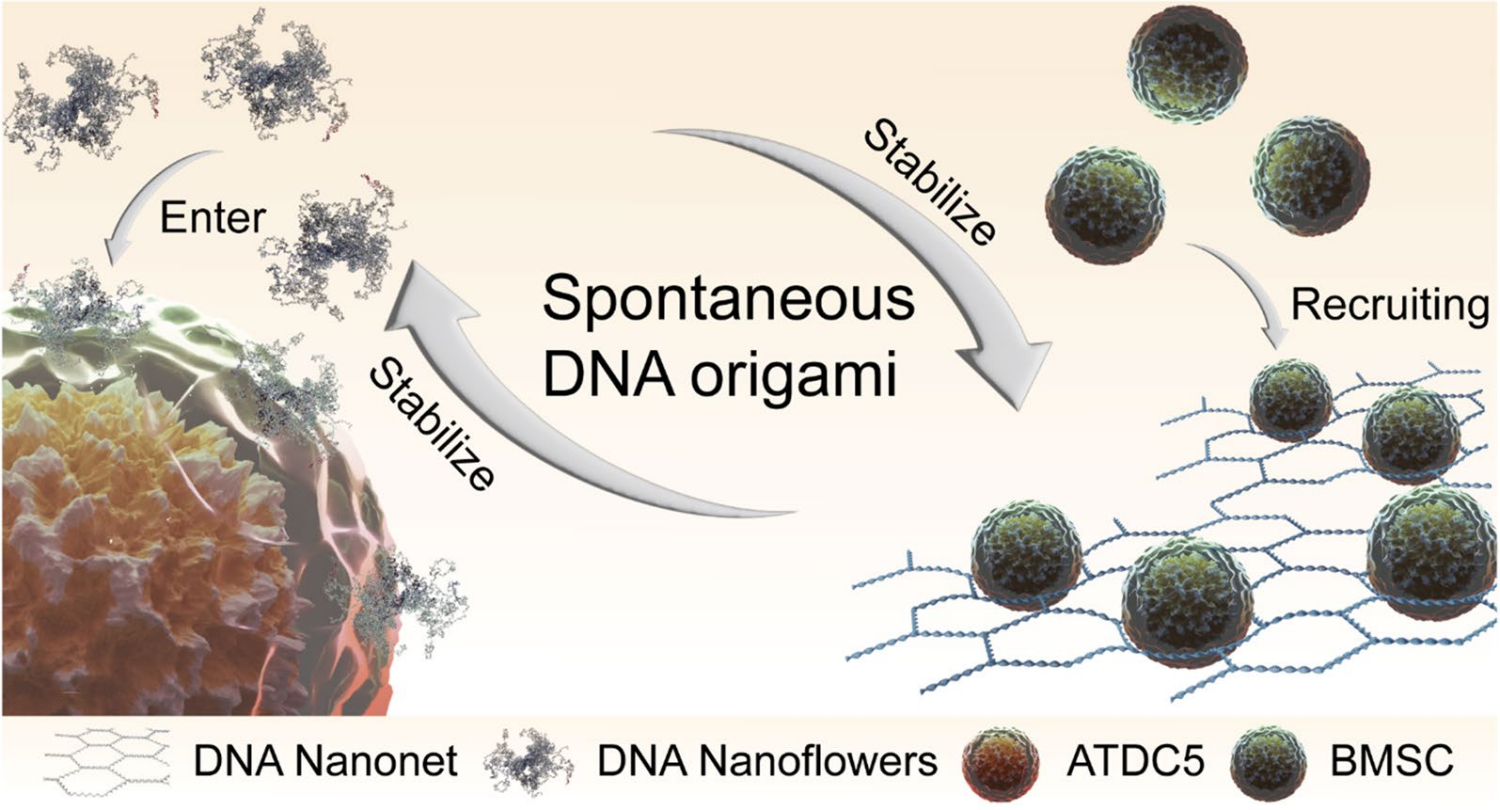
The bimodal DNA self-origami material exhibits properties of spontaneous DNA origami. By controlling the synthesis method, this DNA material can be fixed in two states. Different forms of the material can achieve various functions. DNFs can carry a large amount of small molecular DNA into ATDC5 cells. Functionalized DNNs can recruit several BMSCs

Herein, we propose a spontaneous DNA origami method to construct DNA nanomaterials using ordered DNA materials. We employed oxDNA to conduct coarse-particle simulations of ordered DNA materials composed of three strands. Our results revealed the intriguing phenomenon of spontaneous polymerization and unfolding exhibited by these materials in solution. Subsequently, we constructed a bimodal DNA self-origami material capable of forming DNN and DNF structures. The morphology of this bimodal material changed with the length of the synthetic template chain. By controlling the synthesis time of the template chain, we stabilized the material in a highly ordered and stable form. After freeze-drying, pure DNN and DNF structures existed in the form of molecular crystals, persisting for over 6 months. This nanomaterial could be used to achieve various functions by adjusting the nanoscale DNA structure (Scheme 1). We believe that this material holds immense potential for biomedical tissue engineering applications.

## Methods

### Coarse-particle computer simulation using oxDNA

To explore the formation process and functional characteristics of DNA with various structures, we employed the oxDNA model and version 3.5 of the oxDNA simulation software to conduct molecular dynamics simulations on RTX4080. The parameters used were as follows: steps = 20,000,000, dt = 0.005 ps, thermostat = Langevin, and diff_coeff = 2.5. Subsequently, the results were exported to Blender for visualization. Root mean square fluctuation (RMSF) was used to assess the structural strength of DNA binding (Additional file 1: Fig. S1).

### Synthesis of circular DNA templates

All DNA strands were purchased from Sangon Biotech (Shanghai) Co., Ltd. (the sequences are shown in Additional file 1: Tables S1–S3). A mixture of four DNA short strands was cooled from 95 °C to 37 °C at a rate of 2 °C per 2 min and reacted at 37 °C for 10 min. T4 DNA ligase was added, and the reaction to prepare a solution of circular DNA templates was performed at 16 °C for 10 h (Additional file 1: Figs. S2–S4).

### Synthesis of DNNs

The reaction mixture comprised 4 nM circular DNA template, 20 nM rolling circle amplification (RCA) primer, 56 nM dNTPs (New England), and 4 U Bst enzyme mixture (New England). The reaction was performed at 62 °C for 48 h (Additional file 1: Fig. S5a). Subsequently, three DNA long-chain solutions were mixed in equal proportions. The mixture was cooled from 95 °C to 37 °C at a rate of 2 °C per 2 min and reacted at 37 °C for 10 min for the self-assembly of DNA long chains into pre-DNNs without a topological structure. DNNs with a stable topological structure were obtained by reacting with DNA topoisomerase I (New England) at 37 °C for 15 min.

### Synthesis of a DNN/DNF mixture

The reaction mixture comprised 2 nM circular DNA template, 10 nM RCA primer, 14 nM dNTPs (New England), and 2 U Bst enzyme mixture (New England). The reaction was performed at 62 °C for 24 h (Additional file 1: Fig. S5b). Subsequently, three DNA long-chain solutions were mixed in equal proportions. The mixture was cooled from 95 °C to 37 °C at a rate of 2 °C per 2 min and maintained at 37 °C for 10 min to obtain a DNN/DNF mixed solution.

### Synthesis of DNFs

The reaction mixture comprised 1 nM circular DNA template, 5 nM RCA primer, 3.5 nM dNTPs (New England), and 1 U Bst enzyme mixture (New England). The reaction was performed at 62 °C for 12 h (Additional file 1: Fig. S5c). Subsequently, three DNA long-chain solutions were mixed in equal proportions, and MgCl_2_ solution was added to achieve a final concentration of 10 nM. This step was followed by cooling from 95 °C to 37 °C at a rate of 2 °C per 2 min and reaction at 37 °C for 10 min for the self-assembly of DNA long chains into pre-DNFs without a topological structure. DNFs with a stable topological structure were obtained by reacting with DNA topoisomerase I (New England) at 37 °C for 15 min.

### Purification of DNNs and DNFs

DNNs and DNFs were purified by removing soluble impurities using three times the liquid volume of isopropanol. The mixture of isopropanol, DNNs, and DNFs was incubated at − 20 °C for 20 min and centrifuged at 12,000 rpm for 5 min. Then, the supernatant was removed, and the mixture was freeze-dried in vacuum to obtain a pure DNN and DNF powder (Additional file 1: Fig. S6a, b).

### Freeze–fracture electron microscopic imaging

The samples (2 µL) were dropped onto the microscope stage and rapidly frozen by immersion in liquid nitrogen. Subsequently, the samples were fractured under vacuum and sublimated at − 30 °C for 15 min. Finally, the samples were observed at − 80 °C using focused ion beam scanning electron microscopy at an accelerating voltage of 200 kV.

### Atomic force microscopy (AFM) imaging of DNNs and DNFs

DNNs and DNFs were diluted to 20 nM with Tris–HCl buffer, and 10 μL of the diluted solution was mixed with an equal volume of 10 mM MgCl_2_. The resulting mixture was then dropped and dispersed on a newly peeled mica plate and blow-dried for 15 min on a clean bench. These samples were scanned using an AFM instrument.

### Cell culture

This study was approved by the PLA Ethics Committee. Bone marrow mesenchymal stem cells (BMSCs) were obtained from newborn Sprague–Dawley rats. Phosphate-buffered saline (PBS) (Sigma, USA) containing 0.5% penicillin/streptomycin was used to wash the bilateral femur and tibia; moreover, the bone marrow cavity was washed under sterile conditions to obtain bone marrow fluid. The bone marrow solution was diluted with PBS at a ratio of 1:1, transferred to a single-cell suspension, and centrifuged at 600 rpm for 5 min. The precipitated bone marrow was slowly added to a Percoll separation solution (1.073 g/mL; Pharmacia, NYC, USA) and centrifuged at 1000 rpm for 15 min. Subsequently, the cells were washed with PBS and centrifuged at 600 rpm for 15 min. The cells were then resuspended in low glucose Dulbecco’s modified Eagle’s medium (L-DMEM; Gibco, Waltham, MA, USA) containing 10% fetal bovine serum (FBS; Gibco) and 1% double antibiotic (HyClone) and cultured at 37 °C under 5% CO_2_. When the confluence of the original BMSCs reached approximately 90% of the bottom of the culture flask, the cells were digested with 0.25% trypsin/0.02% ethylenediamine tetraacetic acid (Gibco) at 37 °C and then subcultured at a ratio of 1:3. The second-generation BMSCs were used in follow-up experiments. ATDC5 cells derived from mouse chondrocytes were cultured in L-DMEM (Gibco, Waltham, MA, USA) containing 10% FBS; Gibco) and 1% double antibiotic (HyClone) at 37 °C under 5% CO_2_. The medium was changed every alternate day.

### Zeta point and dynamic light scattering (DLS)

A Malvern Zetasizer Nano S90 system was used to measure the electrophoretic mobility and DLS of DNFs. Laser Doppler velocimetry was performed to determine the zeta potential for assessing the electrophoretic mobility of charged particles. In DLS measurements, the speed of Brownian motion depends on the particle size and viscosity of the medium. The Zetasizer software was used to calculate the size of DNF particles, which reflects the average hydrodynamic diameter and particle strength. The values from three runs are presented as the average ± standard deviation.

### Cell proliferation assay

Cell Counting Kit-8 (CCK-8; Dojindo, Japan) assays were employed to quantitatively measure the proliferation of BMSCs, DNNs, and ATDC5 DNFs cultured for 1, 3, and 7 days. Briefly, the cells were incubated in the CCK-8 working solution according to the manufacturer’s instructions for 2 h at 37 °C. The absorbance of the test solution was determined using a microplate reader (Beckman, Fullerton, CA; n = 6 per group).

### Cell fluorescence experiment.

A fluorescent probe (6 nM) was mixed with a 6-carboxyfluorescein (FAM) label and 1 nM DNFs to obtain a DNF-containing fluorescent probe. DNFs and the fluorescent probe were then mixed with ATDC5 cells and incubated for 30 min, followed by fixation with 2.5% glutaraldehyde. The fixed cells were treated with a 0.5% Triton X-100 solution at 25 °C for 30 min and then washed thrice with PBS for 5 min. Subsequently, the fixed cells were incubated with TRITC Phalloidin (Solarbio) at room temperature for 30 min, washed thrice with PBS for 5 min, and rinsed thrice. This step was followed by incubation with 4′,6-diamidino-2-phenylindole solution (1:500) for 5 min at room temperature for nuclear staining. The cells were then washed thrice with PBS for 5 min. Samples were sealed before image acquisition using a turntable confocal super-resolution imaging system (Olympus SpinSR10).

### Cell-targeted recruitment assay

APT19s (6 nM) and DNNs (1 nM) were mixed at a ratio of 1:6 to obtain functional DNNs (Additional file 1: Fig. S9). Subsequently, 50 μL of the functional DNN solution or an equal concentration of the APT19S solution was dripped onto and dispersed in the central area of each culture dish, and the DNA structure was fixed on the culture dish via ultraviolet irradiation for 1 min. Then, 1 mL of a 1.05 × 10^5^ cells/mL BMSC suspension (in serum-free DMEM) was added to each culture dish, and images were acquired using a light microscope after incubation for 24 h. A gentian violet dye solution was used for cell staining.

### Enhancing the recruitment of nucleic acid aptamers with DNNs

BMSCs were inoculated in the upper chamber of a transwell system, whereas PBS, APT19s, and functional DNNs were placed in the lower chamber. After 24 h of culture, the BMSCs were collected, and a gentian violet dye solution was used for cell staining (Additional file 1: Fig. S10).

## Results

### Molecular simulation of a bimodal DNA self-origami material

**Fig. 1:**
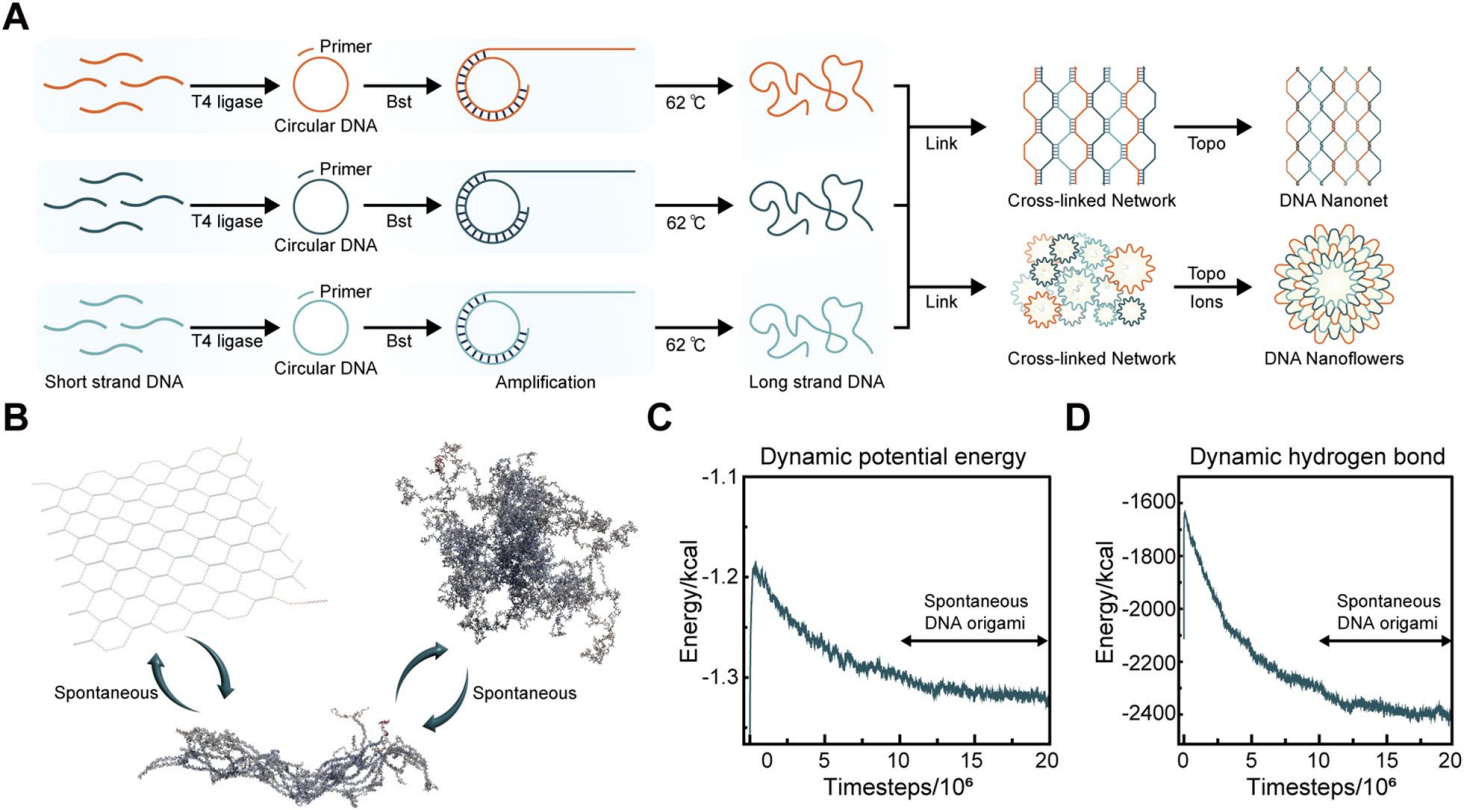
Preparation and molecular dynamics simulation of DNA bimodal materials. **A** Process used for the synthesis of DNNs and DNFs. The synthesized cross-linked network of DNA long chains exhibited two morphologies. One tended to form a DNA nanonetwork under the action of a topological enzyme, whereas the other tended to form a nanoflower structure under the action of ions and a topological enzyme. **B** Morphology of DNNs in the oxDNA simulation. In the simulation of oxDNA, the long-chain DNA exhibited three morphologies: DNN, a bimodal state, and DNF. **C** Dynamic potential energy. During the process of spontaneous DNA origami formation, the dynamic potential energy remained unchanged. **D** Dynamic hydrogen bond. During the process of spontaneous DNA origami formation, the dynamic hydrogen bond remained unchanged

The DNNs and DNFs were composed of long DNA chains. The DNA long chains synthesized via RCA were cross-linked to form pre-DNNs and pre-DNFs. Pre-DNNs were transformed into DNNs with a topological structure under the action of topoisomerase. DNFs were formed under the influence of ions and topoisomerase (Fig. 1A). As the sequence of RCA was repetitive and cyclic, to further explore the process of forming DNA with different structures, we simulated the DNA long chains in four cycles using oxDNA. The results showed that the DNA long chains exhibited two equilibrium states in the same system (Fig. 1B). The dynamic potential energy and hydrogen bonding demonstrated that the potential energy varied only slightly during the hydrogen-bonded recombination and DNA self-origami process (Fig. 1C, D), implying the presence of two DNA materials at equilibrium in the same system. Additional file 1: Fig. S1 presents the RMSF and root mean square deviation.

### Liquid and dry characterization of the bimodal DNA self-origami material

**Fig. 2:**
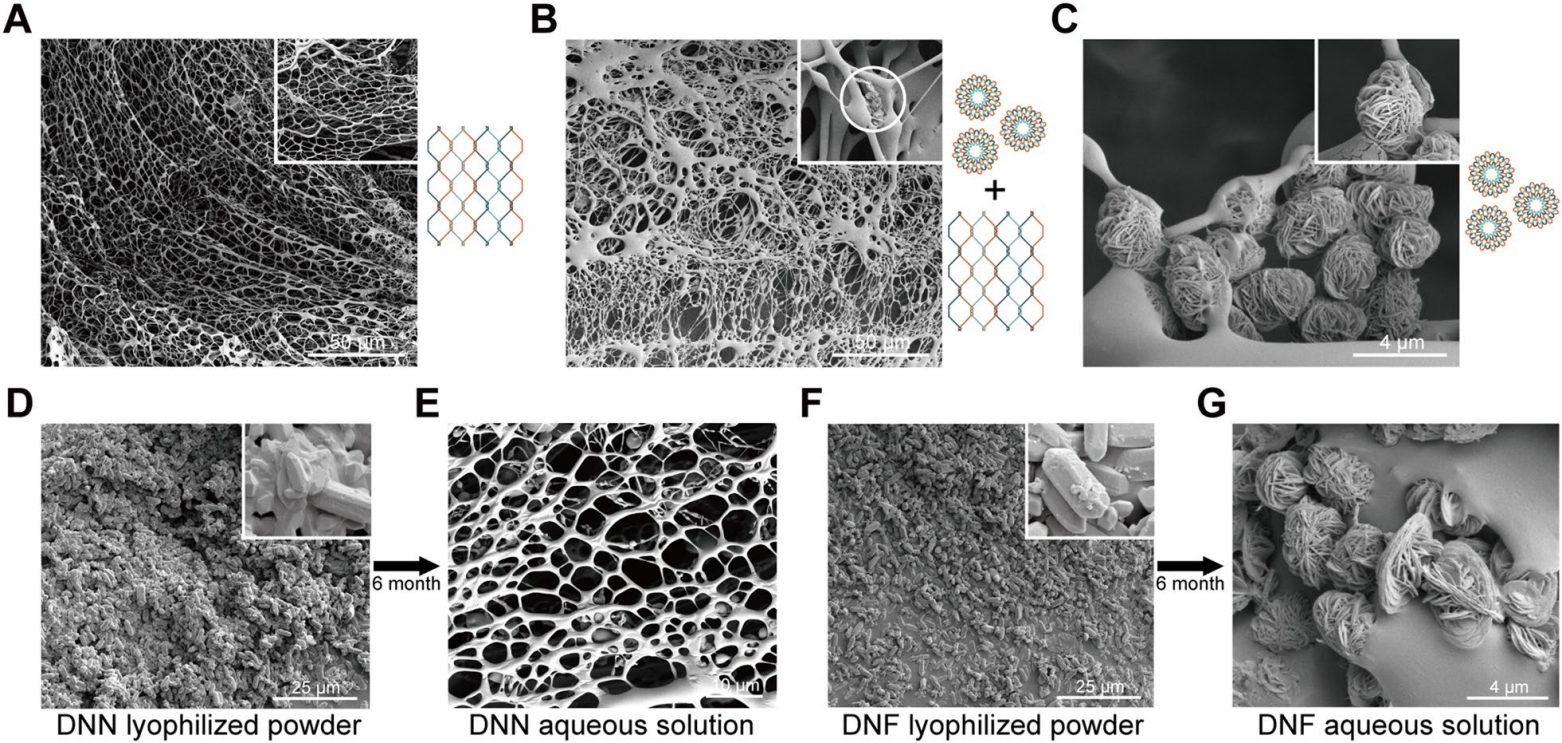
Freeze–fracture electron micrograph of DNA bimodal materials and micrographs taken before and after storage for 6 months. **A** Freeze–fracture electron micrograph of pure DNNs. The DNNs exhibited a wide distribution range, with no formation of DNFs. **B** Freeze–fracture electron micrograph of the DNN and DNF mixture. DNNs had a wide range of distribution, and nanoflowers were embedded in the gap of the net. **C** Freeze–fracture electron micrograph of pure DNFs. DNFs were observed exclusively under the microscope, with the complete absence of DNNs. **D** DNNs in solid state after lyophilization. The dried DNN powder was present in the form of a crystal. **E** DNNs dissolved in water after 6 months. The DNN structure remained intact. **F** DNFs in solid state after lyophilization. The dried DNF powder was present in the form of a crystal. **G** DNFs dissolved in water after 6 months. The DNF structure remained intact

**Fig. 3:**
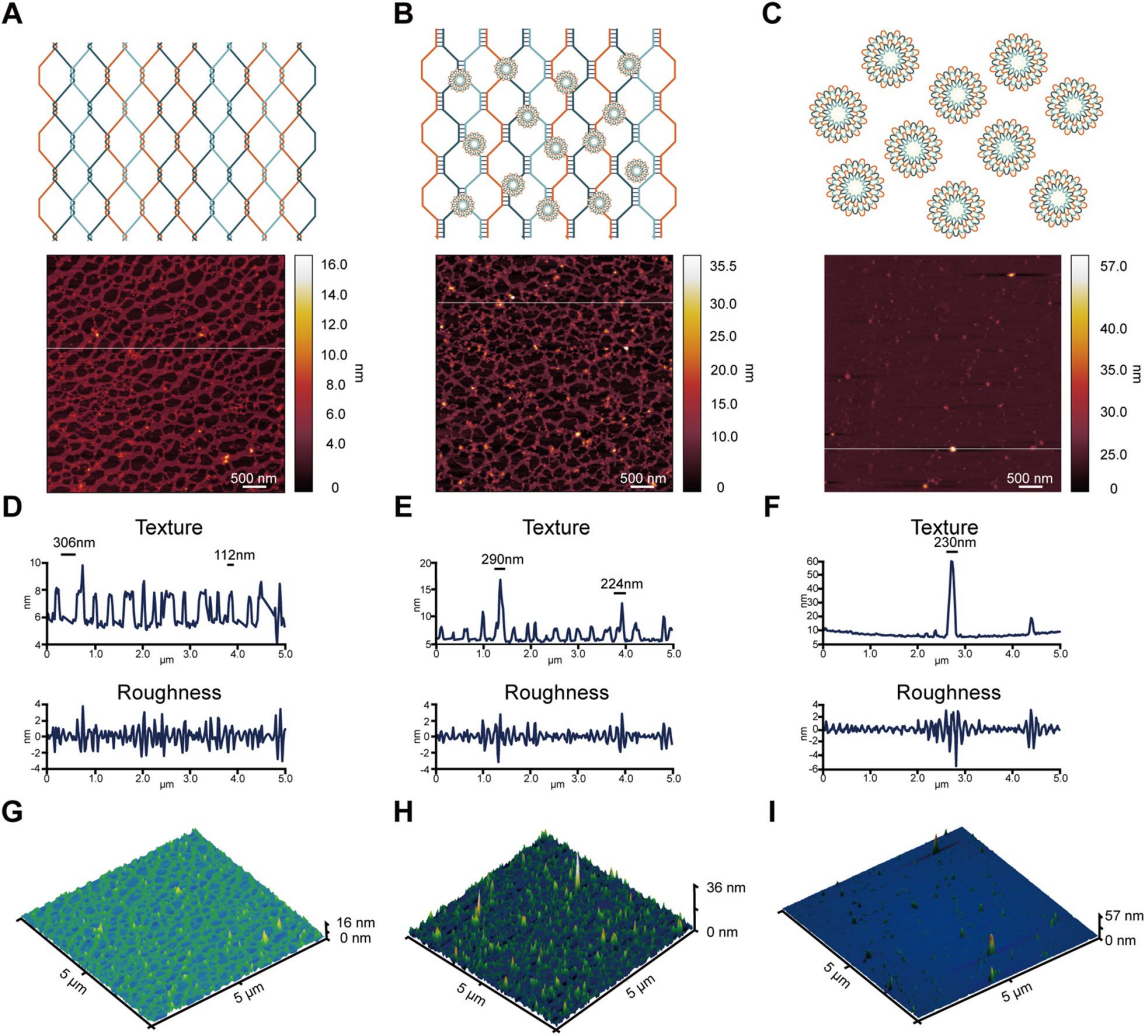
Characterization of bimodal DNA materials in a dry state using AFM. **A** AFM image of pure DNNs (scale: 500 nm). **B** AFM image of coexisting DNNs and DNFs (scale: 500 nm). **C** AFM image of pure DNFs (scale: 500 nm). **D** Quantitative analysis of AFM images of pure DNNs. **E** Quantitative analysis of AFM images with the coexistence of DNNs and DNFs. **F** Quantitative analysis of AFM images of pure DNFs. **G** 3D morphology of AFM images of pure DNNs. **H** 3D morphology of AFM images with the coexistence of DNNs and DNFs. **I** 3D morphology of AFM images of pure DNFs

Using freeze–fracture electron microscopy, we observed that DNNs displayed wide extensibility, covering a large area and exhibiting a uniform pore size without DNF formation (Fig. 2A). We used freeze–fracture electron microscopy to examine the bimodal materials and noted the coexistence of balls and nets. DNFs were embedded on the edge of their pores (Fig. 2B). In the pure DNF solution, there was no DNN formation around pure DNFs, and there were obvious boundaries between DNFs (Fig. 2C). Subsequently, we purified and lyophilized liquid DNNs and DNFs. Using electron microscopic observation, we discerned that DNNs and DNFs existed in the form of molecular crystals (Fig. 2D, F). Moreover, after dissolving DNNs and DNFs that had been lyophilized for 6 months in ddH_2_O, we found that they retained their original form and structure (Fig. 2E, G). Next, to further assess the size of DNNs and DNFs, we performed atomic force microscopic characterization. We observed pure DNNs (Fig. 3A), the coexistence of DNNs and DNFs (Fig. 3B), and pure DNFs (Fig. 3C). Their texture, roughness, and 3D landscape are presented in Fig. 3D–I.

### Enhancement of the cell uptake of a cell fluorescence localization probe by DNFs

**Fig. 4:**
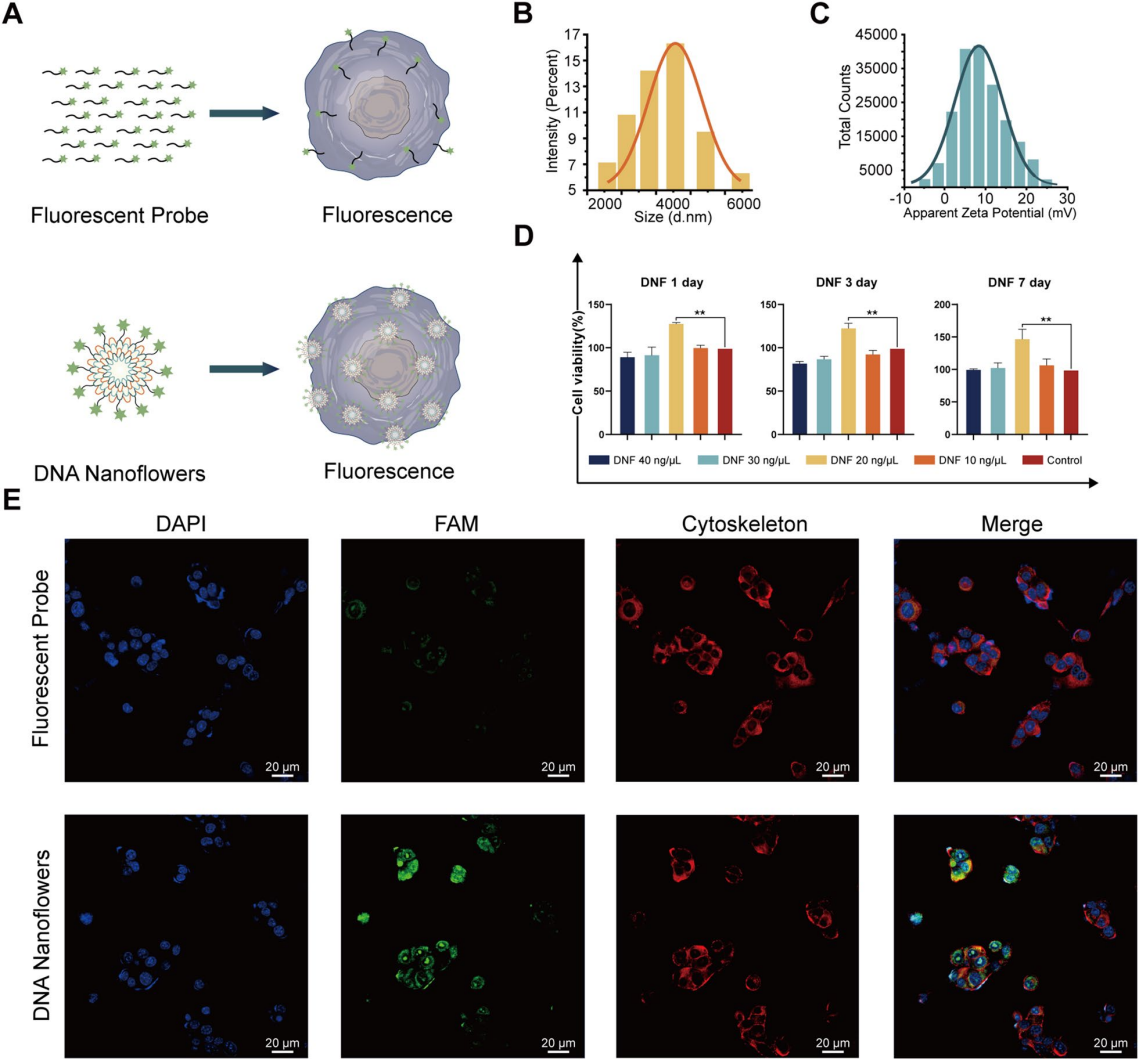
DNA bimodal materials improve the delivery efficiency of small DNA molecules into cells in the form of nanoflowers. **A** Schematic diagram of the fluorescent probe and DNFs entering ATDC5 cells. **B** DLS particle size analysis of DNFs with a FAM fluorescent probe. **C** Zeta potential analysis of DNFs with a FAM fluorescent probe. **D** Identification of DNF cell compatibility. **E** Immunostaining images of DNFs with FAM fluorescence and pure FAM fluorescence in cells. Nucleus (blue), FAM (green), cytoskeleton (red). Scale: 20 μm

Preset binding sites were observed for a significant number of DNA probes on the DNFs, allowing them to carry multiple DNA probes into the cells simultaneously (Fig. 4A). The size of the DNF particles carrying a DNA probe was found to be 4313 nm (Fig. 4B, C). The cell proliferation and cytotoxicity assay experiment demonstrated that a DNF concentration of 20 ng/µL was optimal for carrying probes into cells (Fig. 4D). Using an ultra-micron–resolution imaging system, we observed that DNFs could carry a relevant number of probes into the cells. Within 30 min, a detectable fluorescence signal was observed in the cytoplasm, which was significantly higher than that of the probe-only group (Fig. 4E).

### Targeted recruitment of BMSCs by functional DNNs

**Fig. 5:**
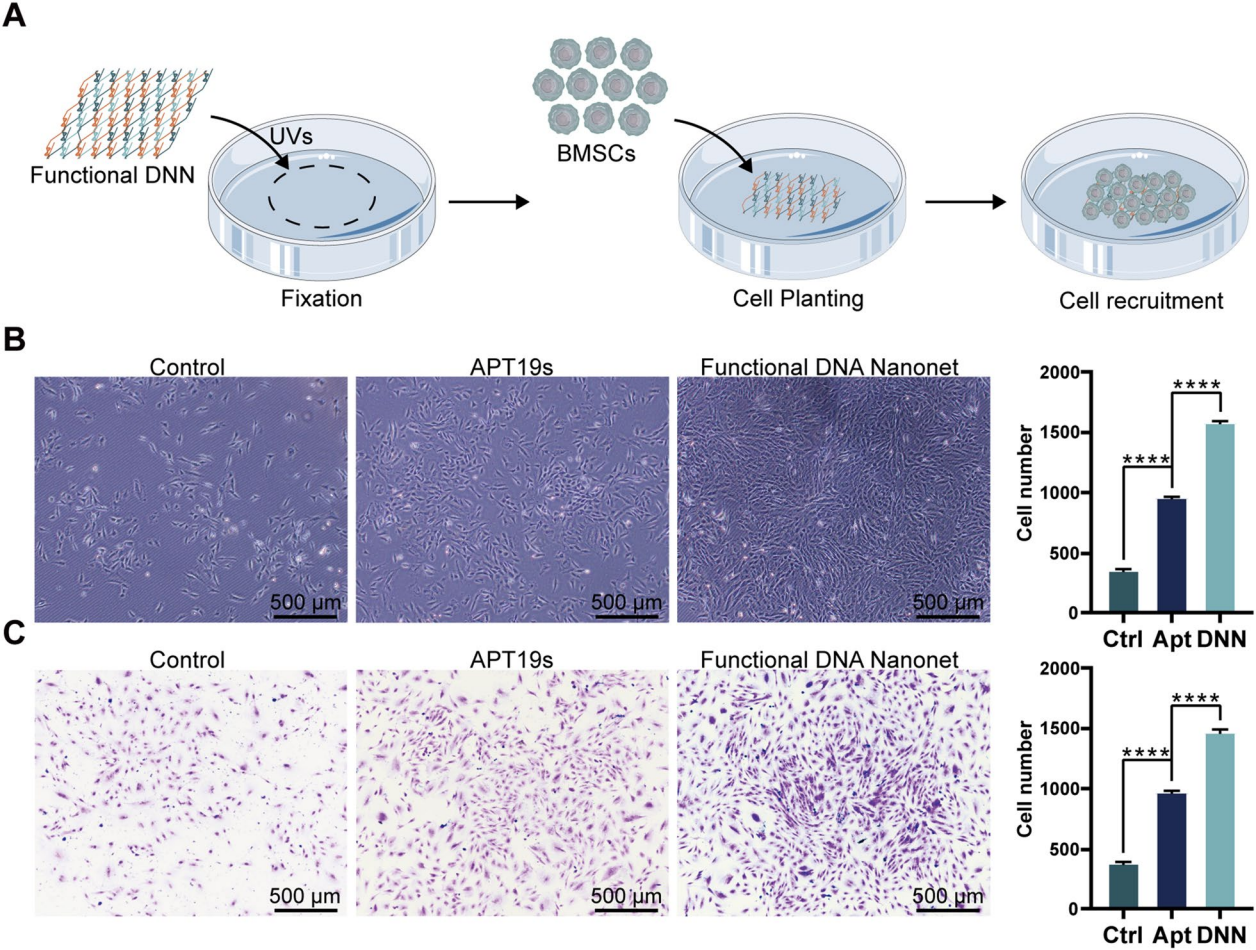
DNA bimodal materials enhance the targeted and efficient recruitment of BMSCs in the form of nanonets. **A** Schematic diagram of functional DNN-targeted BMSCs. The functionalized DNNs were fixed in the center of the Petri dish using ultraviolet irradiation and then incubated with BMSCs; BMSCs were recruited to the functionalized DNN position. **B** Representative images and quantitative analysis of BMSCs recruited by functional DNNs using light microscopy. **C** Crystal violet staining image and quantitative analysis of BMSCs recruited by functional DNNs (n = 5, ****represents significance at P < 0.0001)

We mixed Apt19s and DNNs at a ratio of 6:1 to attach the adapter onto the network and assemble the functional DNNs. Subsequently, we immobilized the functionalized DNNs and an equal concentration of APT19s in an area with a diameter of 1 cm at the center of the culture dish using ultraviolet irradiation (Fig. 5A). Observations were conducted using light microscopy and crystal violet staining (Fig. 5B, C). We found that the effect of recruiting stem cells to the fixed DNA region and functional DNNs was significantly greater than that of APT19s. The CCK-8 kit was used to assess the cytotoxicity of DNNs. The results showed that a concentration of 20 ng/μL of DNN was suitable for cell recruitment (Additional file 1: Fig. S8).

## Discussion

Molecular dynamics simulation and freeze–fracture electron microscopy are powerful tools for studying nucleic acid molecular systems [33–35]. Molecular dynamics simulations can reduce errors in the DNA microstructure introduced by empirical design. However, immense computational power and time are required to explore the DNA self-assembly process [36, 37]. Therefore, coarse-grained DNA simulation is a suitable method for constructing complex DNA models [38–40]. As one of the coarse-grained DNA simulation technology systems, oxDNA can simulate the self-assembly process of DNA in a specific environment [41]. Moreover, freeze–fracture electron microscopy is a key tool for deconstructing the structure of DNA and providing a comprehensive view of a DNA molecule in a liquid environment [42]. However, the sample preparation and shooting process involved in freeze–fracture electron microscopy are complex, precluding its use in the construction and analysis of several nucleic acid molecules with new structures.

We used oxDNA for coarse-particle simulation and combined it with freeze–fracture electron microscopic characterization to examine the DNA microstructure. Herein, we developed a method for constructing high-dimensional DNA materials using an ordered arrangement of three DNA strands. This method allowed the construction of two DNA high-dimensional materials with completely different morphology and functions. The DNA material with spontaneous origami performance fulfilled various functions in different forms. We preset numerous active sites on DNFs and DNNs, which facilitated the transport of multiple DNA probes. DNFs exhibited good biocompatibility and remarkably increased the number of DNA probes transported into cells. This technique holds great potential for cell-targeted drug delivery and fluorescence imaging. Furthermore, DNNs achieved the targeted recruitment and capture of stem cells without a scaffold, which markedly enhanced the recruitment function of APT19s. This method holds immense value in tissue engineering for in situ repair, cell-specific capture, and recruitment. However, small DNA material may have potential effects on cell function and gene expression. Additionally, the large size of DNA materials may enhance cell adhesion but hinder cell uptake and imaging. These limitations necessitate careful consideration in the design and application of DNA materials. To overcome these challenges and maximize their potential in biological applications, it is essential to employ appropriate strategies and techniques that address or mitigate these effects.

## Conclusions

In summary, we successfully constructed a DNA material capable of spontaneous origami using the ordered structure of three long DNA strands. This material exhibited diverse functions under different morphologies. We used molecular simulation and freeze–fracture electron microscopy to simulate two forms of functional DNA materials. Through oxDNA simulation, we found that this DNA material exhibited the spontaneous origami characteristic in solution. Additionally, the purified DNN and DNF powders demonstrated storage stability for over 6 months. We characterized DNNs, DNFs, and their coexisting states using freeze–fracture electron microscopy and AFM. Subsequently, we functionalized DNFs and DNNs. DNFs could carry several DNA molecules into cells within a short time while maintaining biocompatibility. DNNs achieved the targeted recruitment of stem cells without a scaffold when combined with an aptamer. In general, we combined oxDNA and freeze–fracture electron microscopy to develop a novel DNA material. We believe that this nanometer-scale bimodal DNA self-origami material has a high application potential.

### Supplementary Information

**Additional file 1: Figure S1.** Root mean square fluctuation and root mean square deviation of the bimodal DNA self-origami material. **Figures S2–S4.** Structure diagram of circular DNA templates. **Figure S5.** Diagram of DNNs, bimodal state, and DNF synthesis process. **Figure S6.** Schematic diagrams of DNN (**a**) and DNF (**b**) freeze-drying. **Figure S7.** Freeze–fracture electron micrographs of DNNs, bimodal state, and DNFs. **Figure S8.** DNN cytotoxicity analysis. **Figure S9.** Schematic diagram of functionalized DNN preparation. **Figure S10.** Transwell assay for cell migration. **Figure S11.** Fourteen-day stability test of DNNs and DNFs. **Figure S12.** Unit point fluorescence combination of DNFs. **Tables S1–S6.** DNA sequences.

## Data Availability

The original data are included in the article and supplementary materials. Further inquiries can be directed to the corresponding authors.

## Abbreviations

2D: Two-dimensional
3D: Three-dimensional
AFM: Atomic force microscopy
BMSC: Bone marrow mesenchymal stem cell
CCK-8: Cell Counting Kit-8
DLS: Dynamic light scattering
DNF: DNA nanoflower
DNN: DNA nanonet
FAM: ​6-Carboxyfluorescein
FBS: Fetal bovine serum
L-DMEM: Low glucose Dulbecco’s modified Eagle’s medium
PBS: Phosphate-buffered saline
RCA: Rolling circle amplification
RMSF: Root mean square fluctuation
