# Supplementary material for: Bimodal DNA self-origami material with nucleic acid function enhancement
Source: J Nanobiotechnology. 2024 Jan 26;22:39. doi: 10.1186/s12951-024-02296-9 (PMC10821560; doi:10.1186/s12951-024-02296-9)
Supplement: Supplementary file 1 — Additional file 1: Figure S1. Root mean square fluctuation and root mean square deviation of the bimodal DNA self-origami material. Figures S2–S4. Structure diagram of circular DNA templates. Figure S5. Diagram of DNNs, bimodal state, and DNF synthesis process. Figure S6. Schematic diagrams of DNN (a) and DNF (b) freeze-drying. Figure S7. Freeze–fracture electron micrographs of DNNs, bimodal state, and DNFs. Figure S8. DNN cytotoxicity analysis. Figure S9. Schematic diagram of functionalized DNN preparation. Figure S10. Transwell assay for cell migration. Figure S11. Fourteen-day stability test of DNNs and DNFs. Figure S12. Unit point fluorescence combination of DNFs. Tables S1–S6. DNA sequences. [file 12951_2024_2296_MOESM1_ESM.docx]

Supplementary Information for

**Bimodal DNA Self-origami Material with Nucleic Acid Function Enhancement**

**Bimodal DNA Self-origami Material with Nucleic Acid Function Enhancement**

Songlin He ^1, 2†^, Haotian Deng ^1, 2†^, Peiqi Li ^1, 2†^, Qinyu Tian ^1^, Yongkang Yang ^1, 2^, Jingjing Hu ^1,3^, Hao Li ^1, 2^, Tianyuan Zhao ^1^, Hongkun Ling ^2^, Yin Liu ^2,4*^, Shuyun Liu^1,2*^, and Quanyi Guo^1,2*^

^1^ Institute of Orthopedics, First Medical Center, Chinese PLA General Hospital; Beijing Key Laboratory of Regenerative Medicine in Orthopedics; Key Laboratory of Musculoskeletal Trauma and War Injuries PLA, 28 Fuxing Road, Haidian District, Beijing 100853, China.

^2^ School of Medicine, Nankai University, Tianjin 300071, China.

^3^ Department of Gastroenterology, the Second Medical Center and National Clinical Research Center of Geriatric Diseases 28 Fuxing Road, Haidian District, Beijing 100853, China.

^4^ Nankai University Eye Institute, Nankai University Tianjin 300071, China.

^†^ These authors contributed equally to the work.

^*^ Corresponding authors. E-mail address: liuyin@nankai.edu.cn (Y.Liu), clear_ann@163.com (S. Liu), doctorguo_301@163.com (Q. Guo).


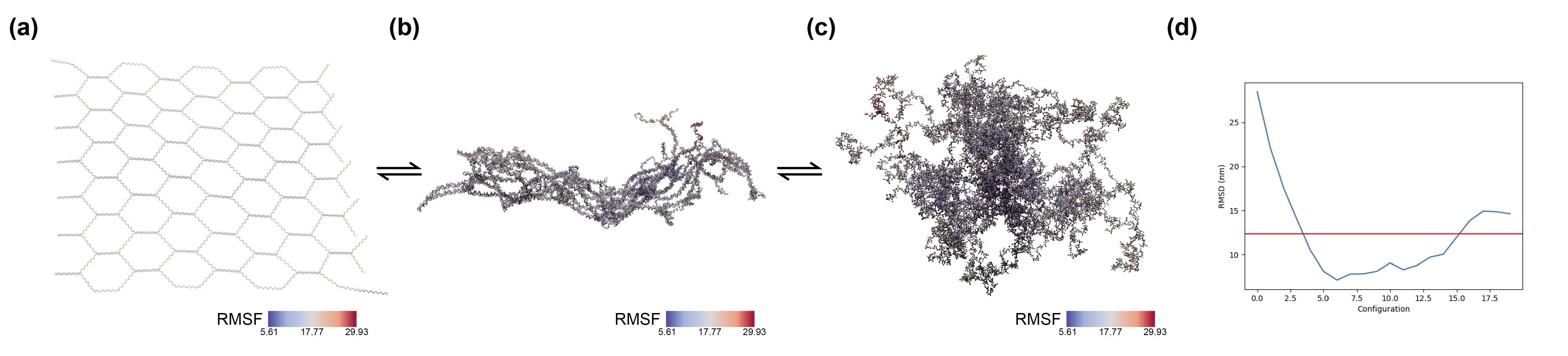


**Figure S1. Root Mean Square Fluctuation (RMSF) and Root Mean Square Deviation (RMSD) of the bimodal DNA self-origami material** (a)RMSF of DNN, (b) RMSF of the intermediate state, (c) RMSF of DNF, (d) the RMSD curve of the self-origami process

**
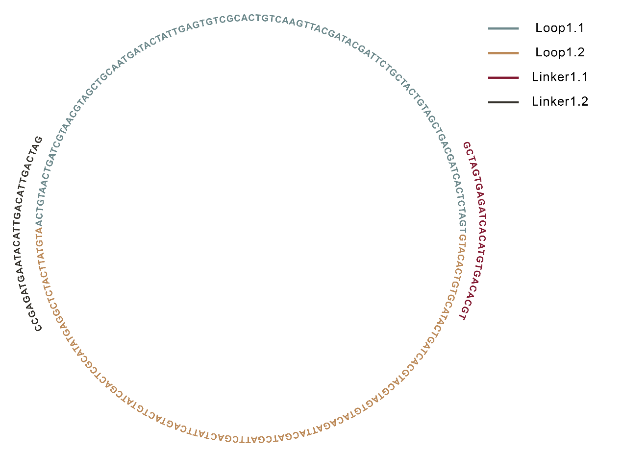
**

**Figure S2. Structure diagram of circular DNA templates (DNA-RCA1)**

**
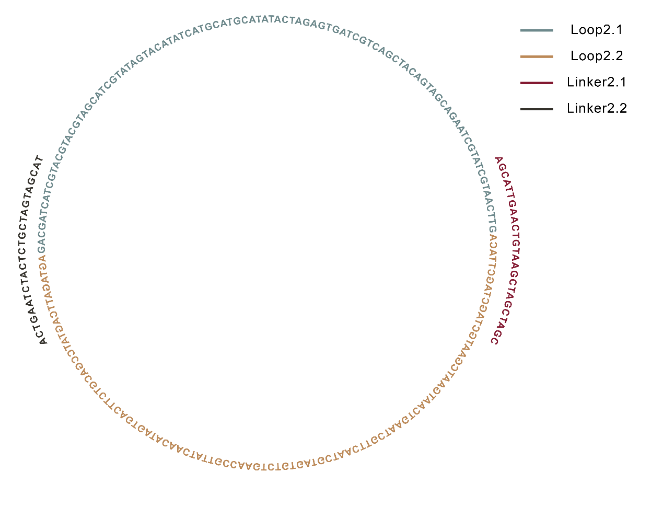
 Figure S3. Structure diagram of circular DNA templates (DNA-RCA2)**

**
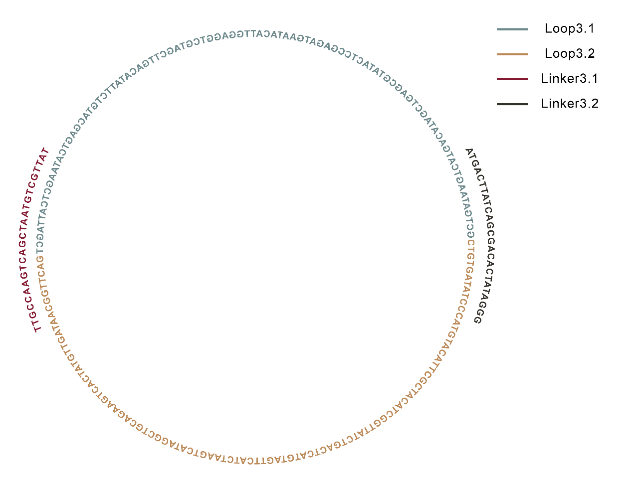
**

**Figure S4. Structure diagram of circular DNA templates (DNA-RCA3)**

**
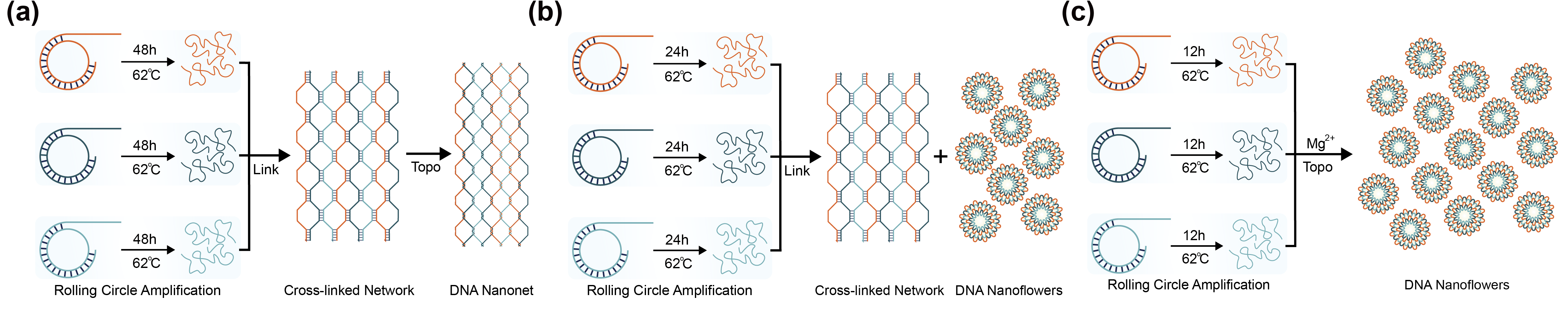
**

**Figure S5. Diagram of DNN(a), bimodal state(b) and DNF(c) synthesizing process.**

**
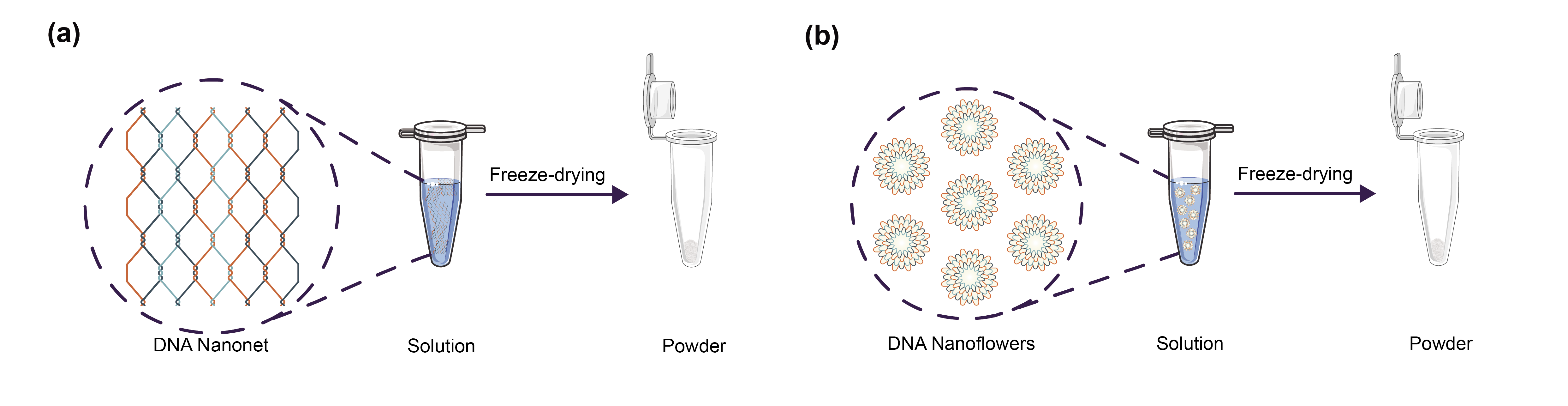
**

**Figure S6.** **Schematic diagram of DNN(a) and DNF(b) freeze drying**

**
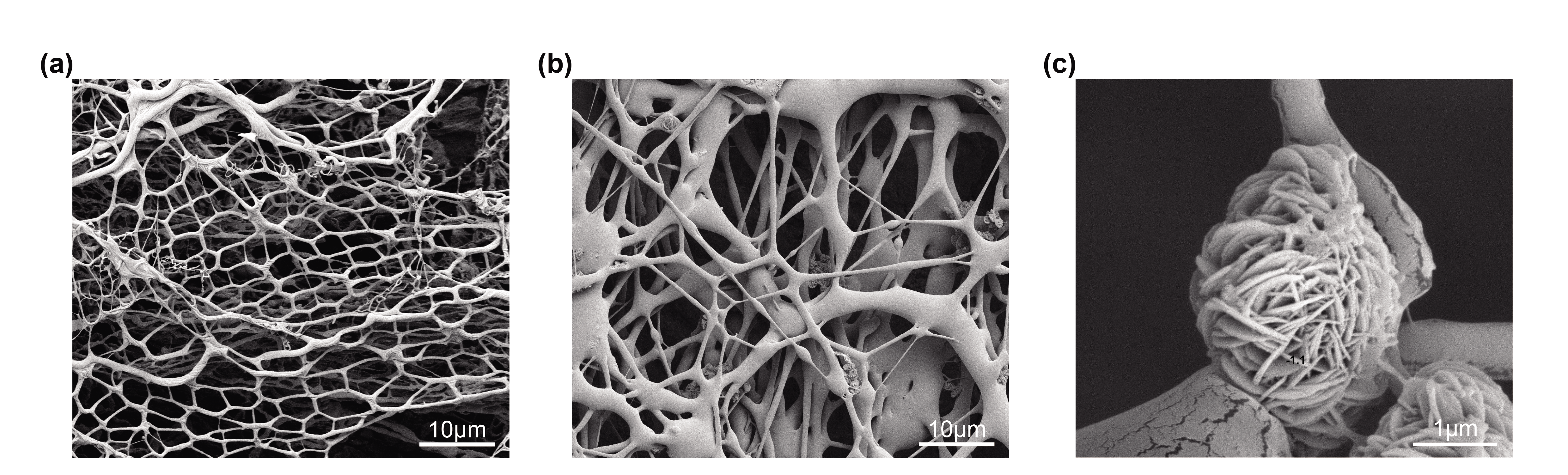
**

**Figure S7.** **Freezing electron microscope images of DNN(a), bimodal state(b) and DNF(c)**


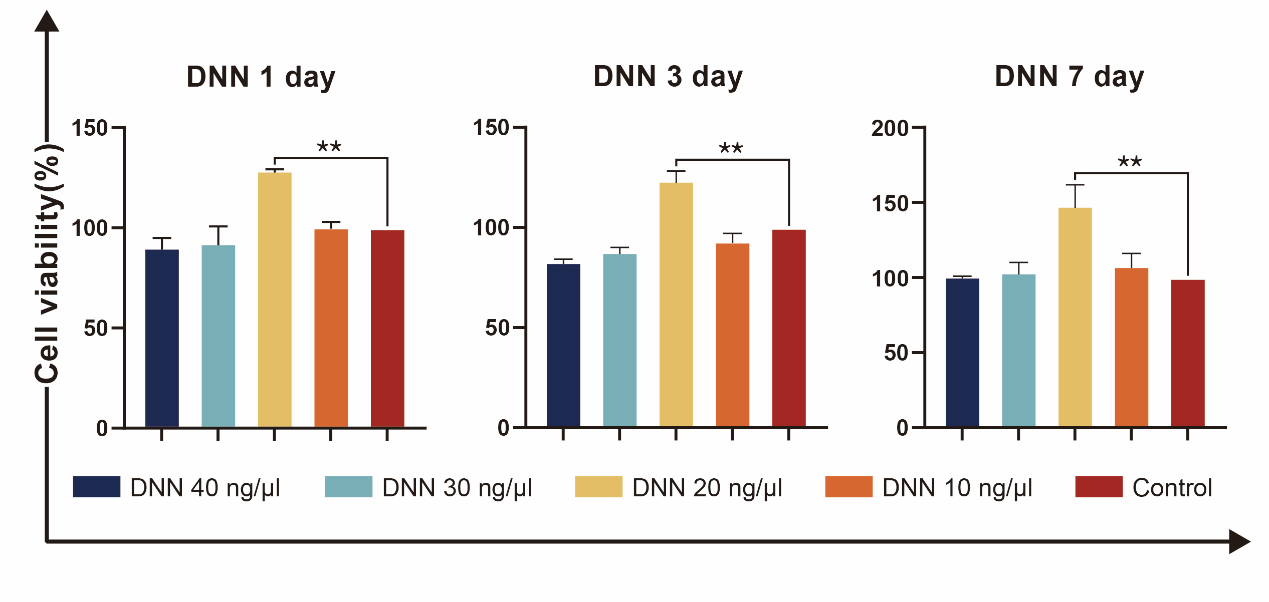


**Figure S8.** **DNN Cytotoxicity Analysis.** Bone marrow stem cells were co incubated with DNN (10 ,20 ,30 ,40 ng/μL) at different time periods (1 day, 3 days, 7 days). The absorbance of the CCK-8 assay was evaluated by microplate reader. The result showed that 20 ng/uL DNN had extremely low cytotoxicity. (n=6 per group, **p<0.01)

**
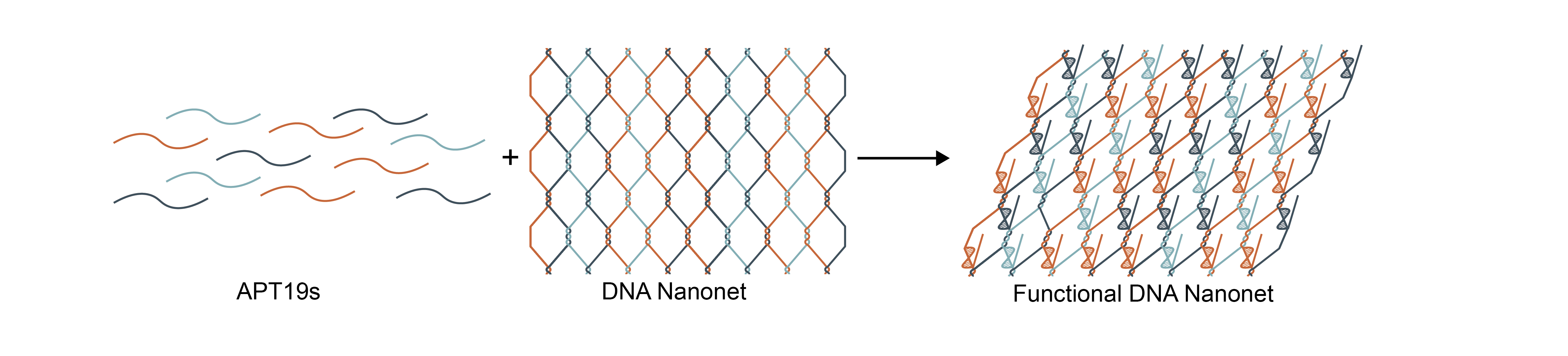
**

**Figure S9.** **Schematic diagram of functionalized DNN preparation.** Functionalized DNN was obtained by mixing 6 nM of APT19s and 1 nM of DNN at a ratio of 1:6 and incubating at room temperature for 1 h.


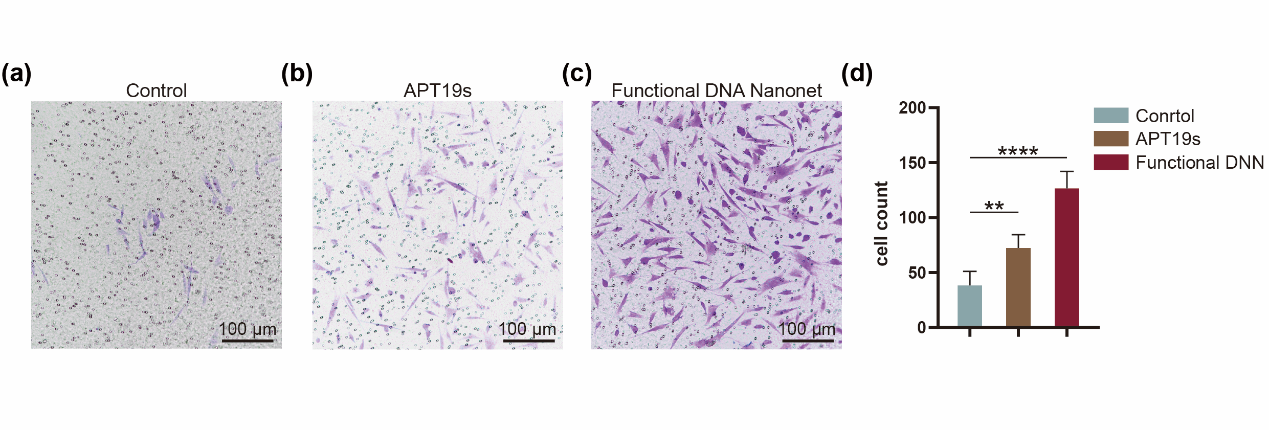


**Figure S10.** **Transwell assay analysis of cell migration.** (a-c) Bone marrow mesenchymal stem cells were placed in the upper chambers of the Transwell system, the lower chambers of the Transwell system were PBS, Apt19s solution and functionalized DNN (20 ng/μL). The migrated cells were stained with crystal violet after incubation for 24 hours. (d) Functionalized DNN can enhance the function of Apt19s aptamer (n=5,** p<0.01，**** p<0.0001)

**
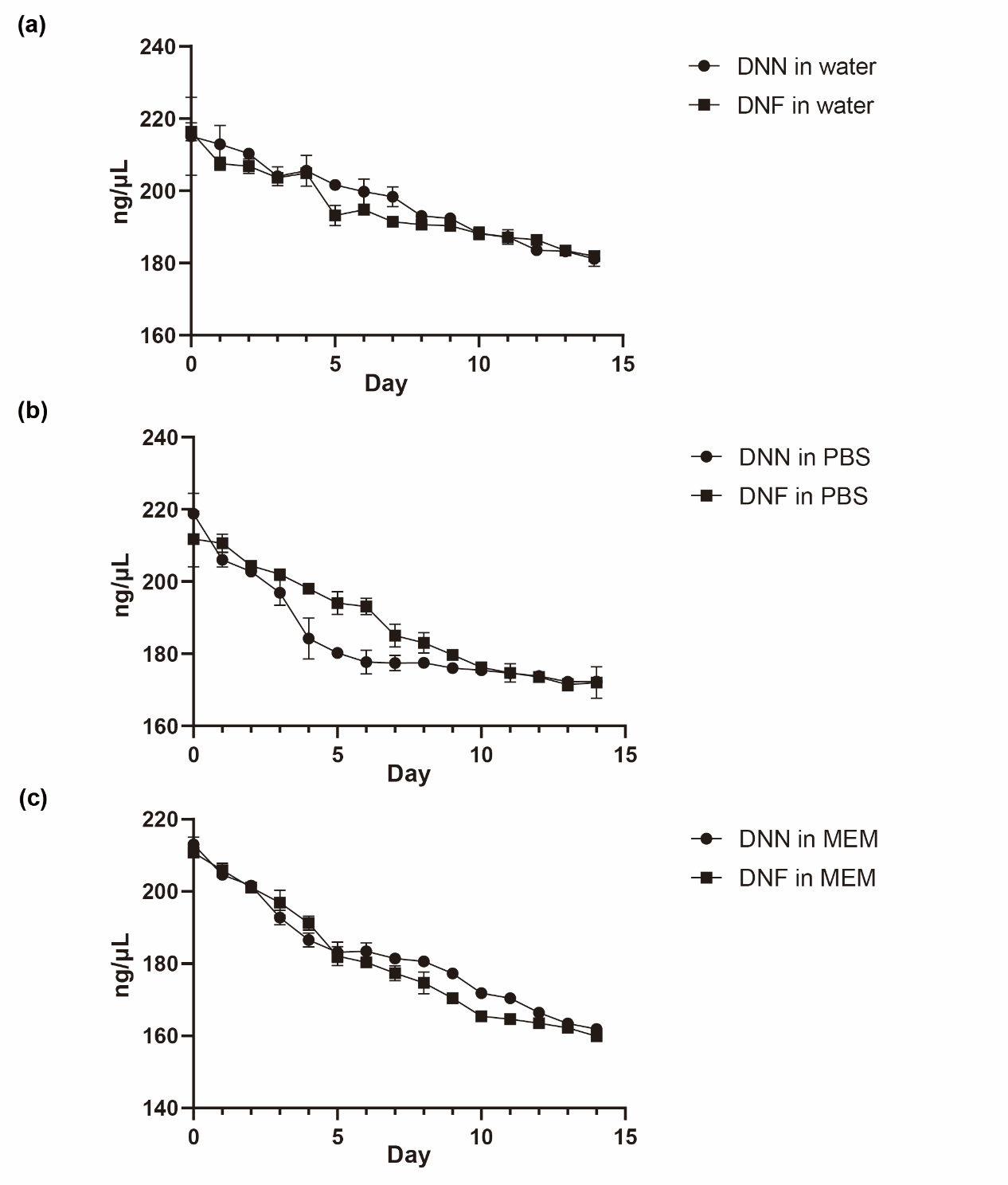
**

**Figure S11.** **14-day stability test of DNN and DNF.** (a) DNN and DNF 14-day stability test in water. (b) DNN and DNF 14-day stability test in PBS. (c) DNN and DNF 14-day stability test in MEM medium.


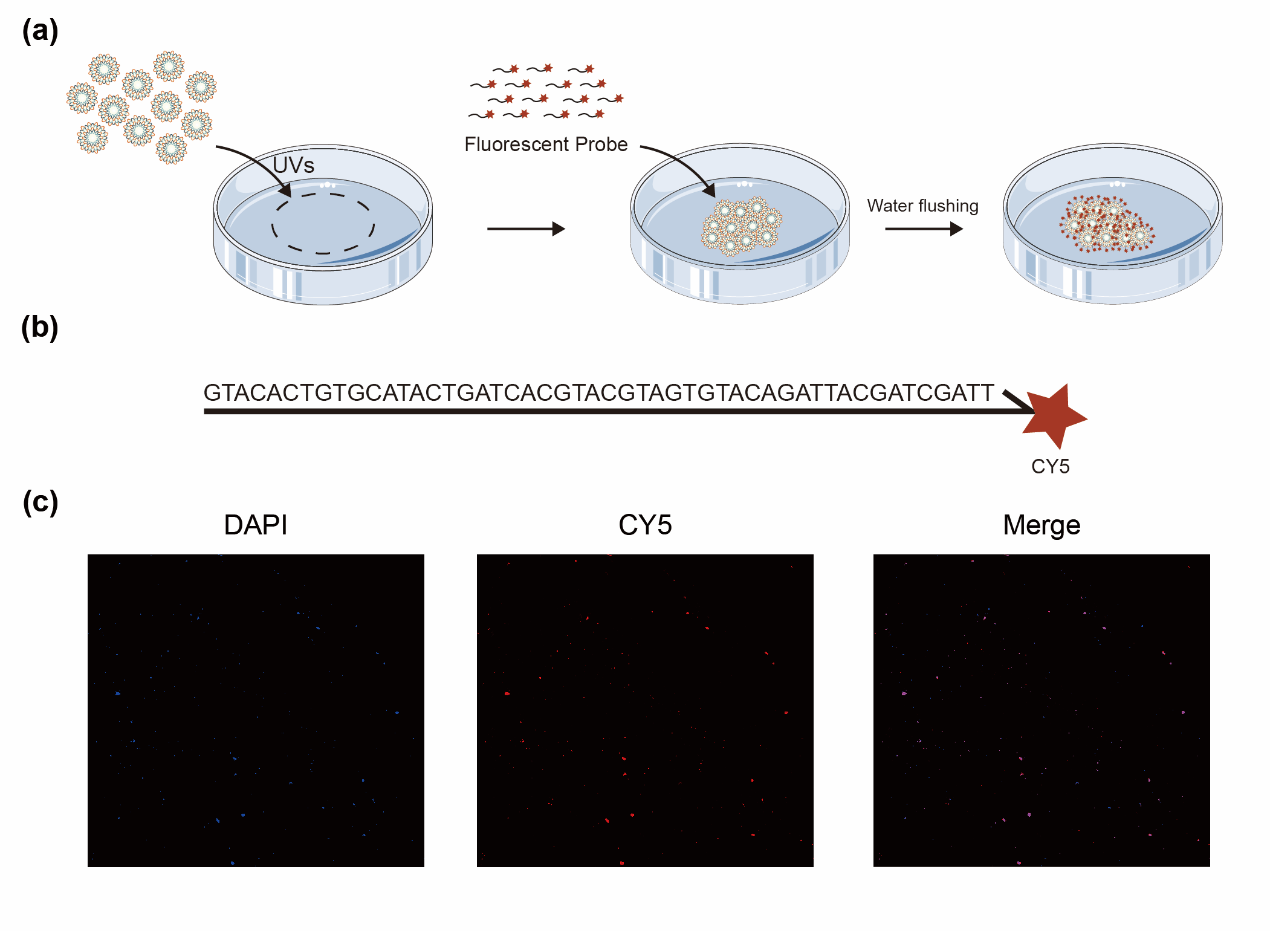


(a) DNF was fixed to a petri dish with UV, incubated with a fluorescent probe for 1h, and then rinsed with water. (b) DNA fluorescent probe sequence. (c) DNN probe combined with fluorescence. DAPI displays the location of the DNN. CY5 shows the position of the fluorescent probe

Table S1. DNA sequences of RCA1

| No. | Sequences Name | Sequences (5’-3’) |
| --- | --- | --- |
| 1 | Loop1.1 | ACTGTAACTGATCGTAACGTAGCTGCAATGATACTATTGAGTGTCGCACTGTCAAGTTACGATACGATTCTGCTACTGTAGCTGACGATCACTCTAGTAT |
| 2 | Loop1.2 | GTACACTGTGCATACTGATCACGTACGTAGTGTACAGATTACGATCGATTCGACTATTCAGTACTGTATCGACTCGCATATGAGGCTCTACTTATGTAAC |
| 3 | Linker1.1 | TGCACAGTGTACACTAGAGTGATCG |
| 4 | Linker1.2 | GATCAGTTACAGTTACATAAGTAGAGCC |
| 5 | Primer1 | AGTGCGACACTCAATAGTATCATTGCAGCT |

Table S2. DNA sequences of RCA2

| No. | Sequences Name | Sequences (5’-3’) |
| --- | --- | --- |
| 1 | Loop2.1 | GACGATCATCGTACGTACGTAGCATCGTATAGTACATATCATGCATGCATATACTAGAGTGATCGTCAGCTACAGTAGCAGAATCGTATCGTAACTTGAC |
| 2 | Loop2.2 | GCTACATTCGATCGATCGTAAGCTAAGTAACTGAATCGTTCAATCGTAGTGTCTGAACCGTTATCAACATAGTGACTTCTGCAGCCTATGACTTAGATGA |
| 3 | Linker2.1 | CGATCGATCGAATGTCAAGTTACGA |
| 4 | Linker2.2 | TACGATGATCGTCTCATCTAAGTCA |
| 5 | Primer2 | ATGCATGCATGATATGTACTATACGATGCT |

Table S3. DNA sequences of RCA3

| No. | Sequences Name | Sequences (5’-3’) |
| --- | --- | --- |
| 1 | Loop3.1 | TCATCGATTACTCGAATACTGAGCATGTCTTATACAGTTCGATGCTGGAGGTTACATAAGTAGAGCCTCATATGCGAGTCGATACAGTACTGAATAGTCG |
| 2 | Loop3.2 | CTGTGATATCCCATGTACATTCGCTACATCGGTTATCTGACTCATGTAGTTCATCTAAGTCATAGGCTGCAGAAGTCACTATGTTGATAACGGTTCAGAC |
| 3 | Linker3.1 | TATTGCTGTAATCGACTGAACCGTT |
| 4 | Linker3.2 | GGGATATCACAGCGACTATTCAGTA |
| 5 | Primer3 | CTCCAGCATCGAACTGTATAAGACATGCTC |

Table S4. DNA sequences of fluorescent probe

| No. | Sequences Name | Sequences (5’-3’) | Modification |
| --- | --- | --- | --- |
| 1 | F1 | GTACACTGTGCATACTGATCACGTACGTAGTGTACAGATTACGATCGATT | FAM |
| 2 | F2 | ACTGTAACTGATCGTAACGTAGCTGCAATGATACTATTGAGTGTCGCACT | FAM |
| 3 | F3 | GCTACATTCGATCGATCGTAAGCTAAGTAACTGAATCGTTCAATCGTAGT | FAM |
| 4 | F4 | GACGATCATCGTACGTACGTAGCATCGTATAGTACATATCATGCATGCAT | FAM |
| 5 | F5 | CTGTGATATCCCATGTACATTCGCTACATCGGTTATCTGACTCATGTAGT | FAM |
| 6 | F6 | TCATCGATTACTCGAATACTGAGCATGTCTTATACAGTTCGATGCTGGAG | FAM |

Table S6. DNA sequences of aptamer

| No. | Sequences Name | Sequences (5’-3’) |
| --- | --- | --- |
| 1 | F1 | GTACACTGTGCATACTGATCACGTACGTAGTGTACAGATTACGATCGATTAGGTCAGATGAGGAGGGGGACTTAGGACTGGGTTTATGACCTATGCGTG |
| 2 | F2 | ACTGTAACTGATCGTAACGTAGCTGCAATGATACTATTGAGTGTCGCACTAGGTCAGATGAGGAGGGGGACTTAGGACTGGGTTTATGACCTATGCGTG |
| 3 | F3 | GCTACATTCGATCGATCGTAAGCTAAGTAACTGAATCGTTCAATCGTAGTAGGTCAGATGAGGAGGGGGACTTAGGACTGGGTTTATGACCTATGCGTG |
| 4 | F4 | GACGATCATCGTACGTACGTAGCATCGTATAGTACATATCATGCATGCATAGGTCAGATGAGGAGGGGGACTTAGGACTGGGTTTATGACCTATGCGTG |
| 5 | F5 | CTGTGATATCCCATGTACATTCGCTACATCGGTTATCTGACTCATGTAGTAGGTCAGATGAGGAGGGGGACTTAGGACTGGGTTTATGACCTATGCGTG |
| 6 | F6 | TCATCGATTACTCGAATACTGAGCATGTCTTATACAGTTCGATGCTGGAGAGGTCAGATGAGGAGGGGGACTTAGGACTGGGTTTATGACCTATGCGTG |
